# Relationship between ventilator-associated pneumonia and mortality in COVID-19 patients: a planned ancillary analysis of the coVAPid cohort

**DOI:** 10.1186/s13054-021-03588-4

**Published:** 2021-05-25

**Authors:** Saad Nseir, Ignacio Martin-Loeches, Pedro Povoa, Matthieu Metzelard, Damien Du Cheyron, Fabien Lambiotte, Fabienne Tamion, Marie Labruyere, Demosthenes Makris, Claire Boulle Geronimi, Marc Pinetonde Chambrun, Martine Nyunga, Olivier Pouly, Bruno Mégarbane, Anastasia Saade, Gemma Gomà, Eleni Magira, Jean-François Llitjos, Antoni Torres, Iliana Ioannidou, Alexandre Pierre, Luis Coelho, Jean Reignier, Denis Garot, Louis Kreitmann, Jean-Luc Baudel, Guillaume Voiriot, Damien Contou, Alexandra Beurton, Pierre Asfar, Alexandre Boyer, Arnaud W. Thille, Armand Mekontso-Dessap, Vassiliki Tsolaki, Christophe Vinsonneau, Pierre-Edouard Floch, Loïc Le Guennec, Adrian Ceccato, Antonio Artigas, Mathilde Bouchereau, Julien Labreuche, Alain Duhamel, Anahita Rouzé, the coVAPid study group, Raphaël Favory, Sébastien Préau, Mercé Jourdain, Julien Poissy, Piehr Saint Leger, Thierry Van der Linden, Anne Veinstein, Elie Azoulay, Frédéric Pene, Maelle Martin, Keyvan Razazi, Gaëtan Plantefeve, Muriel Fartoukh, Didier Thevenin, Bertrand Guidet, Nicolas Weiss, Achille Kouatchet, Charlotte Salmon, Guillaume Brunin, Safaa Nemlaghi, David Meguerditchian, Laurent Argaud, Sebastian Voicu, Charles-Edouard Luyt, Benjamin Kowalski, Edgar Moglia, Luis Morales, Antonia Koutsoukou, Spyros D. Mentzelopoulos, David Nora, Sean Boyd, Julien Maizel, Pierre Cuchet, Quentin Delforge, Jean-Pierre Quenot, Déborah Boyer, Catia Cilloniz

**Affiliations:** 1 Médecine Intensive-Réanimation, CHU de Lille, F-59000 Lille, France; 2 Inserm U1285, CNRS, UMR 8576-UGSF-Unité de Glycobiologie Structurale et Fonctionnelle, Univ. Lille, Lille, France; 3 Department of Intensive Care Medicine, Multidisciplinary Intensive Care Research Organization (MICRO), St. James’s Hospital, St. James Street, Dublin 8, Dublin, Eire Ireland; 4 Hospital Clinic, IDIBAPS, Universided de Barcelona, CIBERes, Barcelona, Spain; 5 Polyvalent Intensive Care Unit, São Francisco Xavier Hospital, Centro Hospitalar de Lisboa Ocidental, and NOVA Medical School, CHRC, New University of Lisbon, Lisbon, Portugal; 6 Center for Clinical Epidemiology and Research Unit of Clinical Epidemiology, OUH Odense University Hospital, Odense, Denmark; 7 Medical ICU, Amiens University Hospital, Amiens, France; 8 Department of Medical Intensive Care, Caen University Hospital, 14000 Caen, France; 9 Service de Réanimation Polyvalente, Centre Hospitalier de Valenciennes, Valenciennes, France; 10 Medical Intensive Care Unit, Rouen University Hospital, Normandie Université, UNIROUEN, Inserm U1096, FHU-REMOD-VHF, 76000 Rouen, France; 11 Department of Intensive Care, François Mitterrand University Hospital, Dijon, France; 12 Intensive Care Unit, University Hospital of Larissa, University of Thessaly, 41110 Biopolis Larissa, Greece; 13 Service de Réanimation Et de Soins Intensifs, Centre Hospitalier de Douai, Route de Cambrai, Douai, France; 14 Service de Médecine Intensive Réanimation, Institut de Cardiologie, Hôpital Pitié-Salpêtrière, Assistance Publique-Hôpitaux de Paris (APHP), Sorbonne Université, 47-83, Boulevard de L’Hôpital, 75651 Paris Cedex 13, France; 15 ICU, Roubaix Hospital, Roubaix, France; 16 Médecine Intensive Réanimation, Hôpital Saint Philibert GHICL, Université Catholique, Lille, France; 17 Réanimation Médicale Et Toxicologique, Hôpital Lariboisière, Université de Paris, INSERM UMRS-1144, Paris, France; 18 Service de Médecine Intensive Et Réanimation, Hôpital Saint-Louis, 1 Avenue Claude Vellefaux, 75010 Paris, France; 19 Critical Care Department, Hospital Universitari Parc Taulí, Sabadell, Spain; 20 1St Department of Intensive Care Medicine, National and Kapodistrian University of Athens Medical School, Evaggelismos Hospital, Athens, Greece; 21 Medical Intensive Care Unit, Cochin Hospital, AP-HP. Centre, Université de Paris, Paris, France; 22 Department of Pulmonology, Hospital Clinic Barcelona, University of Barcelona, IDIBAPS, CIBERES, ICREA, Barcelona, Spain; 23 1St Department of Pulmonary Medicine and Intensive Care Unit, National and Kapodistrian University of Athens, “Sotiria” Chest Hospital, Athens, Greece; 24 Réanimation Polyvalente, CH Lens, Lens, France; 25 Service de Médecine Intensive Réanimation, CHU de Nantes, Nantes, France; 26 Service de Médecine Intensive Réanimation, CHU de Tours, Hôpital Bretonneau, 2 Bd Tonnellé, 37000 Tours, France; 27 Service de Médecine Intensive - Réanimation, Hospices Civils de Lyon, Hôpital Edouard Herriot, 5, place d’Arsonval, 69437 Lyon Cedex 03, France; 28 Service de Médecine Intensive Réanimation, AP-HP, Hôpital Saint-Antoine, Assistance Publique-Hôpitaux de Paris, 184 rue du Faubourg Saint-Antoine, 75571 Paris Cedex 12, France; 29 Sorbonne Université, Assistance Publique-Hôpitaux de Paris, Service de Médecine Intensive Réanimation, Hôpital Tenon, Paris, France; 30 Réanimation Polyvalente, CH Victor Dupouy, Argenteuil, France; 31 Service de Pneumologie, Médecine Intensive - Réanimation (Département “R3S”), AP-HP, Sorbonne Université, Groupe Hospitalier Universitaire Pitié-Salpêtrière Charles Foix, INSERM, UMRS1158 Neurophysiologie Respiratoire Expérimentale Et Clinique, Paris, France; 32 Département de Médecine Intensive-Réanimation, CHU D’Angers, Université D’Angers, 4 rue Larrey, 49933 Angers Cedex 9, France; 33 Intensive Care Unit, Pellegrin-Tripode Hospital, University Hospital of Bordeaux, Bordeaux, France; 34 CHU de Poitiers, Médecine Intensive Réanimation, CIC 1402 ALIVE, Université de Poitiers, Poitiers, France; 35 APHP, CHU Henri Mondor, Service de Médecine Intensive RéanimationUniversité Paris Est-Créteil, Faculté de Santé, Groupe de Recherche Clinique CARMASINSERM U955, Institut Mondor de Recherche Biomédicale, 94010 Créteil, France; 36 Service de Médecine Intensive Réanimation, Centre Hospitalier de Béthune, Réseau de Recherche Boréal, 62408 Béthune, France; 37 Service de Réanimation, Hôpital Duchenne, Rue Monod, 62200 Boulogne-sur-Mer, France; 38 Sorbonne Université, AP-HP, Hôpital de La Pitié-Salpêtrière, Département de Neurologie, Unité de Médecine Intensive Réanimation Neurologique, Paris, France; 39 Intensive Care Unit, Hospital Universitari Sagrat Cor, and Ciber de Enfermedades Respiratorias (Ciberes, CB06/06/0028)-Institut D’Investigacions Biomèdiques August Pi I Sunyer (IDIBAPS), Barcelona, Spain; 40 Critical Care Center, Corporacion Sanitaria Universitaria Parc Tauli, CIBER Enfermedades Respiratorias, Autonomous University of Barcelona, Parc Tauli 1, 08028 Sabadell, Spain; 41 Univ. Lille, CHU Lille, ULR 2694-METRICS: Évaluation Des Technologies de Santé Et Des Pratiques Médicales, 59000 Lille, France

**Keywords:** Ventilator-associated pneumonia, Mortality, COVID-19

## Abstract

**Background:**

Patients with SARS-CoV-2 infection are at higher risk for ventilator-associated pneumonia (VAP). No study has evaluated the relationship between VAP and mortality in this population, or compared this relationship between SARS-CoV-2 patients and other populations. The main objective of our study was to determine the relationship between VAP and mortality in SARS-CoV-2 patients.

**Methods:**

Planned ancillary analysis of a multicenter retrospective European cohort. VAP was diagnosed using clinical, radiological and quantitative microbiological criteria. Univariable and multivariable marginal Cox’s regression models, with cause-specific hazard for duration of mechanical ventilation and ICU stay, were used to compare outcomes between study groups. Extubation, and ICU discharge alive were considered as events of interest, and mortality as competing event.

**Findings:**

Of 1576 included patients, 568 were SARS-CoV-2 pneumonia, 482 influenza pneumonia, and 526 no evidence of viral infection at ICU admission. VAP was associated with significantly higher risk for 28-day mortality in SARS-CoV-2 (adjusted HR 1.70 (95% CI 1.16–2.47), *p* = 0.006), and influenza groups (1.75 (1.03–3.02), *p* = 0.045), but not in the no viral infection group (1.07 (0.64–1.78), *p* = 0.79). VAP was associated with significantly longer duration of mechanical ventilation in the SARS-CoV-2 group, but not in the influenza or no viral infection groups. VAP was associated with significantly longer duration of ICU stay in the 3 study groups. No significant difference was found in heterogeneity of outcomes related to VAP between the 3 groups, suggesting that the impact of VAP on mortality was not different between study groups.

**Interpretation:**

VAP was associated with significantly increased 28-day mortality rate in SARS-CoV-2 patients. However, SARS-CoV-2 pneumonia, as compared to influenza pneumonia or no viral infection, did not significantly modify the relationship between VAP and 28-day mortality.

***Clinical trial registration*:**

The study was registered at ClinicalTrials.gov, number NCT04359693.

**Supplementary Information:**

The online version contains supplementary material available at 10.1186/s13054-021-03588-4.

## Background

Coronavirus disease-19 (COVID-19) pandemic is ongoing, and several million patients have been hospitalized in intensive care units (ICUs) worldwide. A large percentage of COVID-19 patients admitted to the ICU require invasive mechanical ventilation, and are at higher risk for ventilator-associated pneumonia (VAP) [1]. In the large multicenter European coVAPid study [2], SARS-CoV-2 infection was associated with higher risk for VAP, and ventilator associated tracheobronchitis (VAT), as compared to patients with influenza, or no viral infection at ICU admission. Other recent studies [3–7] confirmed these results and reported a high incidence of VAP ranging from 44 to 86%. This could be explained by the prolonged duration of mechanical ventilation in these patients, high rate of acute respiratory distress syndrome (ARDS), common use of corticosteroids, and perhaps specific factors related to severe acute respiratory syndrome Coronavirus 2 (SARS-CoV-2) infection.

Several studies, performed in general ICU populations and using different methods, showed higher mortality rates, and longer duration of mechanical ventilation and ICU stay in VAP patients, as compared with those with no VAP [8–13]. To our knowledge, no study to date has specifically addressed the effect of VAP on mortality in SARS-CoV-2 patients, or the impact of SARS-CoV-2 infection on the relationship between VAP and mortality. Therefore, we conducted this planned ancillary study of the coVAPid European multicenter cohort to determine the impact of VAP on mortality in SARS-CoV-2 patients. We also aimed to determine the impact of SARS-CoV-2 pneumonia, as compared with influenza pneumonia or no viral infection at ICU admission, on the relationship between VAP and mortality.

## Methods

### Study design and population

This study is a planned ancillary analysis of the coVAPid cohort study. Briefly, coVAPid was a multicenter retrospective observational European cohort study. Eligibility criteria included age equal or above 18 years, the need for invasive mechanical ventilation for more than 48 h, and one of the following criteria at ICU admission: (1) SARS-CoV-2 pneumonia, (2) influenza (A or B) pneumonia, or (3) no viral infection.

The Ethics Committee, and Institutional Review Boards approved the study protocol (Comité de Protection des Personnes Ouest VI; approved by April 14, 2020; registration number RIPH:20.04.09.60039) as minimal-risk research using data collected for routine clinical practice, and waived the requirement for informed consent. The study was registered at ClinicalTrials.gov, number NCT04359693.

SARS-CoV-2 infection was confirmed by positive polymerase chain reaction (PCR) testing of a nasopharyngeal or respiratory secretions samples. Influenza pneumonia was diagnosed based on a positive nasopharyngeal or airway secretions PCR test.

### Definitions

#### Ventilator-associated lower respiratory tract infection

The diagnosis of VA-LRTI was based on the presence of at least two of the following criteria: body temperature of more than 38.5 °C or less than 36.5 °C, leucocyte count greater than 12,000 cells per μL or less than 4000 cells per μL, and purulent tracheal secretions [14, 15]. Additionally, all episodes of infection needed microbiological confirmation, with the isolation in the endotracheal aspirate of at least 10^5^ colony-forming units (CFU) per mL, or in bronchoalveolar lavage of at least 10^4^ CFU per mL. VAT was defined with the above-mentioned criteria with no radiographic signs of new pneumonia. VAP was defined by the presence of new or progressive infiltrates on chest X-ray. Only first episodes of VAT and VAP occurring more than 48 h after starting invasive mechanical ventilation were analyzed. All VA-LRTI episodes were prospectively identified, and chest X-rays were reviewed by at least two attending physicians. In case of disagreement, a third physician was asked to interpret the radiograph. Late-onset VAP was defined as VAP diagnosed after 4 days of invasive mechanical ventilation [15]. Initial antibiotic treatment was considered as appropriate when at least one antibiotic, matching the in vitro susceptibility of the pathogen causing VAP, was given to treat this infection [16].

### Outcomes

The primary outcome of this ancillary study was 28-day all-cause mortality. Secondary outcomes included duration of mechanical ventilation, and ICU length of stay censored at 28 days.

### Statistical analysis

Quantitative variables were expressed as medians (interquartile range) and categorical variables were expressed as numbers (percentage). Patient characteristics at ICU admission and during ICU stay were described according to study disease groups (SARS-CoV-2 pneumonia vs. Influenza pneumonia vs. no viral infection) without formal statistical comparisons. Patient’s outcomes (overall survival, mechanical ventilation duration, length of ICU stay) were described according to study disease groups using survival analysis approach by estimating the cumulative incidence of event of interest (death, extubation alive and ICU discharge alive) censored at 28-days. Cumulative incidence of death was estimated using Kaplan–Meier method, cumulative incidence of extubation alive and ICU discharge alive were estimated using Kalbfleisch and Prentice method, considering death as competing event [17].

We assessed the effect of SARS-CoV-2 pneumonia compared to the two other disease groups on patient’s outcomes using a univariable and multivariable marginal Cox’s regression models to account clustered (center) data, with cause-specific hazard for mechanical ventilation duration and length of ICU stay (considering extubation alive and ICU discharge alive as event of interest, and death as competing event). Pre-specified potential predictors of patient outcomes (age, gender, SAPS II, Charlson score, McCabe classification, shock and ARDS) were included as covariates into multivariable models. Hazard ratios (HRs) for SARS-CoV-2 pneumonia vs. Influenza pneumonia, and HRs for SARS-CoV-2 pneumonia vs. no viral infection were derived from Cox’s models as effect size. A HR > 1 indicates a decrease in survival, mechanical ventilation duration and length of ICU stay, whereas a HR < 1 indicates an increase in survival, mechanical ventilation duration and length of ICU stay.

In each disease group, we assessed the association of first episodes of VA-LRTI with patient’s outcomes using univariable and multivariable marginal Cox’s regression models, with cause-specific hazard for mechanical ventilation duration and length of ICU stay and by considering the first VA-LRTI occurrence as a time-dependent covariate (3-levels categorical variable: No VA-LRTI, vs. VAT, vs. VAP); as well as binary variable: No VA-LRTI vs. VA-LRTI (VAT or VAP). This model accounted for exposure time of VA-LRTI, by comparing at each follow-up time point, the current VA-LRTI status of patients who have the event to patients who are at risk (without the event of interest and without the competing event for mechanical ventilation duration and length of ICU stay) [18].

HRs associated with VAT, VAP, and no VA-LRTI were derived as effect size using time period without VA-LRTI as reference. To assess whether the association between VA-LRTI and outcomes differed between the three disease groups, a heterogeneity test was performed [19], comparing the HRs for VA-LRTI (VAP, VAT, VAT + VAP combined) between study groups.

To avoid case-deletion in multivariate analyses due to presence of missing data in covariates, multivariable Cox’s models were performed after handling missing data on covariates by using multiple imputation procedure [20]. This imputation was performed using regression-switching approach (chained equations with m = 20 obtained) under the missing at random assumption using all baseline characteristics (see Table 1), disease group and outcomes (event status and log of event time), with a predictive mean matching method for quantitative variables and logistic regression model (binary, ordinal or multinomial) for categorical variables. Estimates obtained in the different imputed data sets were combined using Rubin’s rules [21].

**Table 1:** Patient characteristics at ICU admission according to disease group, and 28-day mortality

	SARS-CoV-2 pneumonia		Influenza pneumonia		No viral infection	
	Alive (*n* = 402)	Dead (*n* = 166)	Alive (*n* = 350)	Dead (*n* = 132)	Alive (*n* = 344)	Dead (*n* = 182)
Age, years^a^	62 (53–70)	70 (62–78)	61 (52–70)	65 (54–72)	63 (52–72)	70 (60–76)
Men	281/402 (69.9)	126/166 (75.9)	219/350 (62.6)	79/131 (60.3)	239/342 (69.9)	114/182 (62.6)
Body mass index, kg/m^2 b^	28.7 (25.5–33.6)	29.1 (26.0–33.0)	27.5 (23.1–32.3)	27.6 (23.5–31.7)	26.3 (22.7–29.8)	26.9 (23.2–33.3)
Severity scores						
SAPS II^c^	38 (31–51)	48 (38–61)	48 (37–60)	58 (45–72)	51 (39–63)	63 (51–73)
SOFA score^d^	6 (3–8)	7 (4–10)	8 (5–10)	10 (7–13)	8 (5–11)	9 (7–12)
Comorbidity scores						
MacCabe classification						
Non-fatal	347/382 (90.8)	128/161 (79.5)	249/332 (75.0)	75/124 (60.5)	216/318 (67.9)	99/171 (57.9)
Fatal < 5 years	33/382 (8.6)	29/161 (18.0)	78/332 (23.5)	36/124 (29.0)	90/318 (28.3)	47/171 (27.5)
Fatal < 1 year	2/382 (0.5)	4/161 (2.5)	5/332 (1.5)	13/124 (10.5)	12/318 (3.8)	25/171 (14.6)
Charlson Comorbidity Index^e^	2 (1–3)	4 (2–5)	3 (2–5)	4 (2–6)	3 (2–5)	5 (3–6)
Chronic diseases						
Diabetes mellitus	113/399 (28.3)	55/166 (33.1)	72/344 (20.9)	32/130 (24.6)	82/338 (24.3)	50/181 (27.6)
Chronic renal failure	11/395 (2.8)	22/164 (13.4)	25/346 (7.2)	14/129 (10.9)	22/339 (6.5)	23/182 (12.6)
Cardiovascular disease	59/396 (14.9)	44/164 (26.8)	80/346 (23.1)	37/130 (28.5)	72/337 (21.4)	62/181 (34.3)
Chronic heart failure	12/394 (3.0)	9/164 (5.5)	19/345 (5.5)	18/130 (13.8)	24/336 (7.1)	26/182 (14.3)
COPD	26/396 (6.6)	11/164 (6.7)	96/344 (27.9)	33/131 (25.2)	63/339 (18.6)	35/182 (19.2)
Chronic respiratory failure	12/394 (3.0)	8/164 (4.9)	56/344 (16.3)	11/131 (8.4)	28/336 (8.3)	21/182 (11.5)
Cirrhosis	6/395 (1.5)	2/164 (1.2)	10/345 (2.9)	6/130 (4.6)	18/335 (5.4)	18/181 (9.9)
Immunosuppression	29/395 (7.3)	23/164 (14.0)	61/348 (17.5)	46/131 (35.1)	71/340 (20.9)	46/180 (25.6)
Active smoking	20/396 (5.1)	9/164 (5.5)	122/346 (35.3)	27/130 (20.8)	102/337 (30.3)	35 /182 (19.2)
Alcohol abuse	29/394 (7.4)	5/164 (3.0)	65/345 (18.8)	20/130 (15.4)	89/337 (26.4)	43/182 (23.6)
Location before ICU admission						
Home	187/402 (46.5)	84/166 (50.6)	212/349 (60.7)	63/132 (47.7)	180/344 (52.3)	85/182 (46.7)
Hospital ward	154/402 (38.3)	61/166 (36.7)	104/349 (29.8)	53/132 (40.2)	147/344 (42.7)	83/182 (45.6)
Another ICU	61/402 (15.2)	21/166 (12.7)	33/349 (9.5)	16/132 (12.1)	17/344 (4.9)	14/182 (7.7)
Admission category						
Medical	401/402 (99.8)	166/166 (100.0)	348/350 (99.4)	132/132 (100.0)	302/344 (87.8)	165/182 (90.7)
Surgical	0/402 (0.0)	0/166 (0.0)	0/350 (0.0)	0/132 (0.0)	11/344 (3.2)	6/182 (3.3)
Trauma	1/402 (0.2)	0/166 (0.0)	2/350 (0.6)	0/132 (0.0)	31/344 (9.0)	11/182 (6.0)
Recent hospitalization (< 3 months)	26/401 (6.5)	18/165 (10.9)	41/348 (11.8)	31/131 (23.7)	90/342 (26.3)	58/182 (31.9)
Recent antibiotic treatment (< 3 months)	50/402 (12.4)	24/165 (14.5)	56/347 (16.1)	39/130 (30.0)	56/342 (16.4)	47/182 (25.8)
Causes for ICU admission						
Shock	58/394 (14.7)	44/163 (27.0)	144/343 (42.0)	66/127 (52.0)	147/336 (43.8)	97/179 (54.2)
Acute respiratory failure	371/401 (92.5)	150/166 (90.4)	316/349 (90.5)	117/131 (89.3)	196/334 (58.7)	83/179 (46.4)
ARDS	271/398 (68.1)	115/165 (69.7)	157/342 (45.9)	63/127 (49.6)	44/330 (13.3)	28/179 (15.6)
Neurological failure	13/385 (3.4)	13/163 (8.0)	51/339 (15.0)	18/126 (14.3)	128/331 (38.7)	63/178 (35.4)
Cardiac arrest	1/384 (0.3)	2/163 (1.2)	14/338 (4.1)	11/127 (8.7)	40/329 (12.2)	44/179 (24.6)
Acute kidney injury	48/385 (12.5)	48/163 (29.4)	84/337 (24.9)	49/124 (39.5)	87/327 (26.6)	49/178 (27.5)

Values are as no./No. (%) or median (interquartile range)

McCabe classification of comorbidities and likelihood of survival, likely to survive > 5 years, 1–5 years, < 1 year; Chronic renal failure, KDOQI CKD classification stage 4 or 5 (creatinine clearance < 30 ml/mn); Chronic heart failure, NYHA class III or IV; Heart disease, ischemic heart disease or atrial fibrillation; Cirrhosis, Child–Pugh score B or C; Immunosuppression if haematological malignancy, allogenic stem cell transplant, organ transplant, HIV or immunosuppressive drugs; More than one cause for ICU admission is possible

^a^1 missing value in influenza group; ^b^ 160 missing values (SARS-CoV-2, *n* = 32; influenza, *n* = 68; controls, *n* = 60); ^c^ 87 missing values (SARS-CoV-2, *n* = 43; influenza, *n* = 21; controls, *n* = 21); ^d^ 27 missing values (SARS-CoV-2, *n* = 21; influenza, *n* = 4; controls, *n* = 2); ^e^ 50 missing values (SARS-CoV-2, *n* = 19; influenza, *n* = 11; controls, *n* = 20)

Statistical testing was performed at the two-tailed α level of 0.05. Data were analyzed using the SAS software package, release 9.4 (SAS Institute, Cary, NC).

## Results

### Patient characteristics

In total, 1576 patients were included from March 2016 through May 2020 (568 in SARS-CoV2, 482 in influenza, and 526 in no viral infection groups). 399 (25.3%) VAP, and 167 (10.6%) VAT first episodes were diagnosed in study patients. 28-day mortality was 28.8% (164 of 568 patients), 22% (125 of 482 patients), and 32.9% (173 of 526 patients) in SARS-CoV-2, influenza, and no viral infection groups, respectively. Older age, higher SAPS II and SOFA score, comorbidities, septic shock, cardiac arrest, and acute kidney injury rates at ICU admission were more common in non-survivors, as compared to survivors in the 3 study groups. During ICU stay, percentage of patients who received corticosteroids, prone positioning, and ECMO was higher in non-survivors as compared to survivors. The characteristics of study patients are presented in Tables 1, and 2.

**Table 2:** Patient characteristics during ICU stay according to disease groups and 28-day mortality

	SARS-CoV-2 pneumonia		Influenza pneumonia		No viral infection	
	Alive (*n* = 402)	Dead (*n* = 166)	Alive (*n* = 350)	Dead (*n* = 132)	Alive (*n* = 344)	Dead (*n* = 182)
Antiviral treatment	226/401 (56.4)	96/165 (58.2)	319/349 (91.4)	118/132 (89.4)	19/343 (5.5)	5/180 (2.8)
Oseltamivir	30/399 (7.5)	14/164 (8.5)	314/344 (91.3)	116/131 (88.5)	18/342 (5.3)	4/179 (2.2)
Remdesivir	21/399 (5.3)	6/164 (3.7)	0/344 (0.0)	0/131 (0.0)	0/342 (0.0)	0/179 (0.0)
Lopinavir-Ritonavir	96/399 (24.1)	51/164 (31.1)	0/344 (0.0)	0/131 (0.0)	0/342 (0.0)	0/179 (0.0)
Lopinavir-Ritonavir + interferon	12/399 (3.0)	9/164 (5.5)	0/344 (0.0)	0/131 (0.0)	0/342 (0.0)	0/179 (0.0)
Hydroxychloroquine	134/399 (33.6)	39/164 (23.8)	0/344 (0.0)	1/131 (0.8)	0/342 (0.0)	0/179 (0.0)
Corticosteroids	131/380 (34.5)	71/162 (43.8)	124/345 (35.9)	58/130 (44.6)	97/343 (28.3)	64/182 (35.2)
Hydrocortisone	27/377 (7.2)	32/160 (20.0)	64/343 (18.7)	43/130 (33.1)	39/340 (11.5)	41/180 (22.8)
Dexamethasone	32/377 (8.5)	16/160 (10.0)	1/343 (0.3)	0/130 (0.0)	6/340 (1.8)	4/180 (2.2)
Methylprednisolone	70/377 (18.6)	21/160 (13.1)	58/343 (16.9)	15/130 (11.5)	51/340 (15.0)	17/180 (9.4)
Highest daily dose, mg^a^	100 (67–133)	71 (50–133)	75 (50–100)	50 (50–100)	63 (50–100)	50 (50–75)
Duration, days^b^	6 (4–9)	6 (4–8)	5 (3–9)	6 (3–9)	4 (2–7)	4 (3–8)
Antibiotic treatment	363/378 (96.0)	143/153 (93.5)	309/330 (93.6)	125/128 (97.7)	268/323 (83.0)	147/173 (85.0)
Duration, days	7 (5–9)	7 (4–10)	7 (5–11)	7 (4–9)	7 (4–9)	6 (4–9)
Prone positioning	263/401 (65.6)	120/166 (72.3)	96/349 (27.5)	55/132 (41.7)	33/340 (9.7)	30/182 (16.5)
ECMO	39/402 (9.7)	22/165 (13.3)	38/349 (10.9)	22/131 (16.8)	2/341 (0.6)	3/182 (1.6)

Vales are no./No. (%) or median (interquartile range)

ECMO, Extracorporeal Membrane Oxygenation; ICU, Intensive Care Unit; MV, Mechanical Ventilation

^a^11 missing values (SARS-CoV-2, *n* = 4; influenza, *n* = 4; controls, *n* = 3); ^b^16missing values (SARS-CoV-2, *n* = 7; influenza, *n* = 3; controls, *n* = 6)

**Fig. 1:**
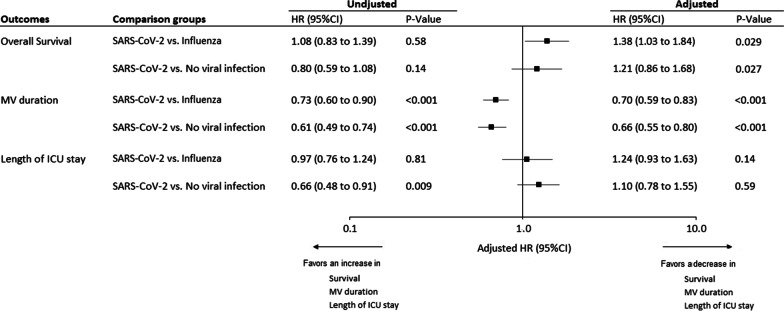
Unadjusted and Adjusted hazard ratios for 28-day mortality, extubation alive and ICU discharge alive, associated with SARS-CoV-2 pneumonia, versus influenza pneumonia and no viral infection groups. HRs were calculated using cause-specific proportional hazard models, by considering mortality as competing event for MV duration, and length of ICU stay. Adjusted HRs were calculated, including age, gender, simplified acute physiology score II, Charlson score, MacCabe classification, shock, and acute respiratory distress syndrome as pre-specified covariates in Cox’s models (after handling missing values by multiple imputation). A HR > 1 indicates a decrease in survival duration (i.e. an increased risk for mortality), MV duration (i.e. an increased risk for extubation alive) and ICU length of stay (i.e. an increased risk for discharge alive) and a HR < 1 indicates an increase in survival duration (i.e. a decreased risk for mortality), MV duration (i.e. a decreased risk for extubation alive) and ICU length of stay (i.e. a decreased risk for discharge alive). Note that the event of interest for survival is a pejorative event (death) whereas for MV duration and ICU length of stay, the event of interest is a positive event (extubation or discharge alive). Consequently, the detrimental effect of SARS-CoV-2 pneumonia (vs influenza pneumonia and no viral infection groups) was associated with a HR > 1 for overall survival but was associated with a HR < 1 for MV duration and ICU length of stay. HR, hazard ratio; ICU, intensive care unit; MV, mechanical ventilation

The cumulative incidence of 28-day mortality, extubation alive, and ICU discharge alive, according to study groups, are presented in Additional file 1: Fig. 1. SARS-CoV-2 infection was associated with significantly longer duration of mechanical ventilation, as compared to influenza and no viral infection groups (Fig. 1). SARS-CoV-2 infection was also associated with significantly higher risk for mortality, as compared to influenza group (Fig. 1).

### Characteristics of patients with ventilator-associated pneumonia

Antimicrobial treatment was prescribed to 191 of 205 (93.2%), 98 of 105 (93.3), and 82 of 87 (94.3) patients with VAP in SARS-CoV-2, influenza, and no viral infection groups; respectively. Initial antibiotic treatment was appropriate in 145 of 200 (72.5%), 69 of 102 (67.7%), and 54 of 87 (62.1%) VAP patients in SARS-CoV-2, influenza, and no viral infection group; respectively. Median (interquartile range) time from starting invasive mechanical ventilation to VAP occurrence was 9 (6, 13) days in SARS-CoV-2, 9 (5, 13) days in influenza, and 7 (4, 12) days in no viral infection groups. Percentage of patients with late-onset VAP was 82.4% (169 of 200 patients), 73.8% (79 of 102 patients), and 65.5% (57 of 87 patients) in SARS-CoV-2, influenza, and no viral infection group; respectively. *Pseudomonas aeruginosa*, Enterobacter spp., and Klebsiella spp. were the most frequently identified microorganisms (Table 3). Percentage of VAP patients with MDR was lower in SARS-CoV-2 patients as compared to the two other groups, as well as percentage of patients with late-onset VAP related to MDR (35 of 167 patients (21%), 27 of 78 patients (34.6%), and 20 of 57 patients (35.1%), in SARS-CoV-2, influenza, and no viral infection groups; respectively).

**Table 3:** Microorganisms responsible for ventilator-associated pneumonia

	SARS-CoV-2 pneumonia (*n* = 205)	Influenza pneumonia (*n* = 107)	No viral infection (*n* = 87)
Gram-positive cocci			
MSSA	20 (9.8)	5 (4.7)	8 (9.2)
MRSA	6 (2.9)	4 (3.7)	2 (2.3)
Enterococcus spp.	7 (3.4)	2 (1.9)	2 (2.3)
* Streptococcus pneumoniae*	7 (3.4)	1 (1)	2 (2.3)
Streptococcus spp.	1 (0.5)	0 (0)	0 (0)
Other	0 (0)	2 (1.9)	3 (3.5)
Gram-negative bacilli			
* Pseudomonas aeruginosa*	51 (24.9)	26 (24.3)	15 (17.2)
Enterobacter spp.	37 (18)	15 (14)	12 (13.8)
Klebsiella spp.	26 (12.7)	17 (15.9)	12 (13.8)
* Escherichia coli*	19 (9.2)	8 (7.5)	5 (5.7)
* Acinetobacter baumannii*	9 (4.4)	16 (15)	10 (11.5)
* Stenotrophomonas maltophilia*	4 (2)	2 (1.9)	4 (4.6)
* Serratia marcescens*	9 (4.4)	4 (3.7)	1 (1.1)
* Citrobacter freundii*	6 (2.9)	1 (1)	1 (1.1)
Citrobacter spp.	5 (2.4)	3 (2.8)	3 (3.5)
* Proteus mirabilis*	5 (2.4)	1 (1)	2 (2.3)
* Haemophilus influenza*	3 (1.5)	5 (4.7)	5 (5.7)
* Morganella morganii*	2 (1)	3 (2.8)	0 (0)
Other	26 (12.7)	8 (7.5)	5 (5.7)
Polymicrobial	24 (11.7)	8 (7.5)	6 (6.9)
Multidrug-resistant isolates*	42 (20.7)	42 (40)	27 (31)

Data are presented as *N* (%)

^*^Missing data: 2, 2 in SARS-CoV-2 and Influenza groups; respectively

MRSA, methicillin-resistant *Staphylococcus aureu*s; MSSA, methicillin-sensitive *Staphylococcus aureus*

### Primary and secondary study outcomes

**Fig. 2:**
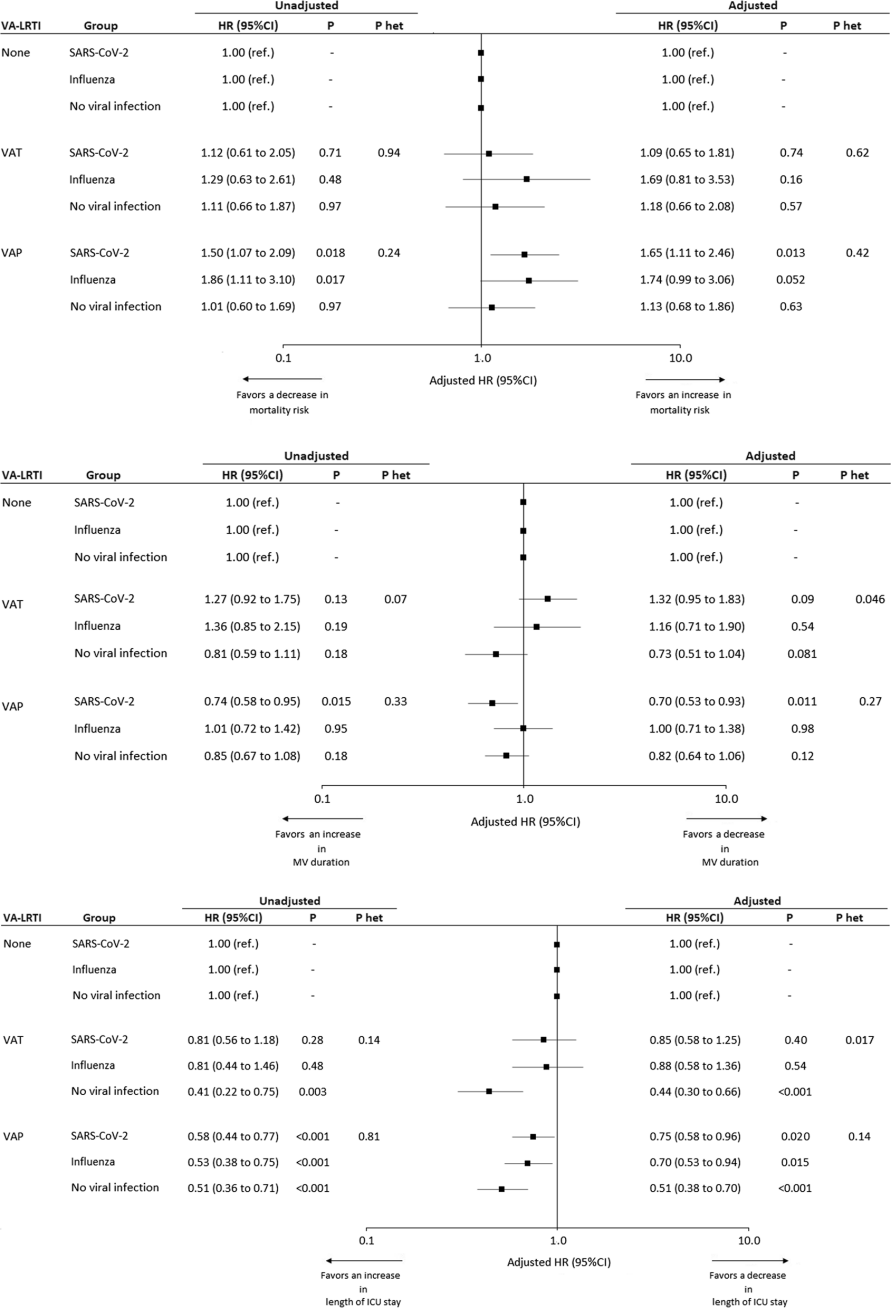
Association between ventilator-associated lower respiratory tract infections and outcomes. **a** 28-Day mortality. **b** Duration of mechanical ventilation. **c** Length of ICU stay. HRs were calculated using cause-specific proportional hazard models, considering the first VA-LRTI as a time dependent 3-levels categorical variable (No VA-LRTI vs. VAT vs. VAP). Adjusted HRs were calculated including age, gender, simplified acute physiology score II, Charlson score, MacCabe classification, shock, and acute respiratory distress syndrome as pre-specified covariables in Cox’s model. Since the event of interest for 28-Day mortality is a pejorative event (death), whereas for MV duration and ICU length of stay, the event of interest is a positive event (extubation or discharge alive), the detrimental effect of SARS-CoV-2 pneumonia (vs influenza pneumonia and no viral infection groups) was associated with a HR > 1 for 28-Day mortality, with a HR < 1 for MV duration and ICU length of stay. HR, hazard ratio; ICU, intensive care unit; MV, mechanical ventilation; VA-LRTI, ventilator-associated respiratory tract infection; VAP, ventilator-associated pneumonia; VAT, ventilator-associated tracheobronchitis

VAP was associated with significantly higher risk for 28-day mortality in SARS-CoV-2, and influenza groups, but not in no viral infection group (Fig. 2A). However, the heterogeneity test showed no significant difference in the strength of association between VAP and mortality across the 3 study groups.

VAP was associated with significantly longer duration of mechanical ventilation in the SARS-CoV-2 group, but not in influenza or no viral infection groups. The heterogeneity test showed no significant difference between the 3 groups regarding the association between VAP and duration of mechanical ventilation (Fig. 2B).

VAP was associated with significantly longer duration of ICU stay in all study groups. The heterogeneity test showed no significant difference between the 3 groups regarding the association between VAP and ICU length of stay (Fig. 2C).

VAT was not significantly associated with increased risk for mortality, or longer duration of mechanical ventilation in the 3 study groups. VAT was associated with significantly longer ICU stay in no viral infection group, but not in the two other groups. The heterogeneity test was significant between the 3 groups regarding duration of ICU stay (Fig. 2a–c).

Descriptive characteristics and outcomes of patients with, or without, VA-LRTI are presented in Additional file 1: Tables 1, and 2. The association between VA-LRTI and outcomes, according to study groups, is presented in Additional file 1: Fig. 2.

## Discussion

### Main results

The main results of our study are that VAP is associated with increased 28-day mortality rate and longer duration of mechanical ventilation and ICU length of stay in SARS-CoV-2 patients. SARS-CoV-2 infection, compared with influenza or no viral infection, has no significant impact on the relationship between VAP and 28-day mortality, neither on the relationship between VAP and duration of mechanical ventilation, or ICU length of stay.

### Relationship between VAP and mortality in SARS-CoV-2 patients

Few data are available on mortality rate in SARS-CoV-2 patients with VAP. Although several recent studies evaluated the incidence of VAP in these patients, only one study performed in ARDS patients requiring ECMO [6], reported on mortality rate (30%) in this population. However, this study was performed in a single center, the number of patients with SARS-CoV-2 infection was small (*n* = 50), and no comparison was performed with mortality rate in SARS-CoV-2 patients with no VAP.

Previous studies, performed in general ICU populations, showed an increased mortality rate in VAP patients [22–25]. A large meta-analysis was performed on individual data from 6284 patients included in randomized controlled trials of VAP prevention [8]. The overall attributable mortality of VAP was 13%, with higher rates for surgical patients and patients with a mid-range severity score at admission. Attributable mortality was mainly caused by prolonged exposure to the risk of dying due to increased length of ICU stay. However, other studies suggested that mortality attributable to VAP was small [26, 27].

### Impact of SARS-CoV-2 infection on the relationship between VAP and mortality

Multidrug resistant bacteria (MDR) bacteria and inappropriate initial antibiotic treatment are well-known risk factors for mortality in VAP patients [28, 29]. Although the incidence of MDR, and inappropriate initial antibiotic treatment was lower in COVID-19 patients, as compared to the two other groups, the relationship between VAP and mortality was only significant in COVID-19 patients. This suggests that SARS-CoV-2 infection and specific pulmonary lesions might play a role in the severity and outcome of VAP in this population. Nevertheless, the absence of significant heterogeneity between the three groups suggests that SARS-CoV-2 infection has no significant impact on the relationship between VAP and mortality. At least three explanations could be provided for this result. First, potential confounders might have influenced our results. However, careful adjustment was performed on SAPS II, age, gender, comorbidities, septic shock, and ARDS, based on the results of prior studies [13, 30]. Second, the number of patients with VAP was relatively small in the no viral infection group, and the study might have been underpowered to detect a significant effect. Third, SARS-CoV-2 infection could be associated with similar impact on the relationship between VAP and mortality. Previous studies suggested that COVID-19 patients have similar outcomes as other patients with similar type of acute illness. For example, a recent study reported a 90-day mortality rate of 37% in COVID-19 ARDS [1], which is in line with previous results in non-COVID ARDS [31].

### Strengths and limitations

To our knowledge, our study is the first to evaluate the relationship between VAP and mortality in SARS-CoV-2 patients. Strengths of this study are the large number of included patients, the multicenter design, and the two control groups including patients with influenza pneumonia or no viral infection. In addition, we carefully adjusted for potential confounders, using competing risk analyses, and Kalbfleisch and Prentice methods. Cox’s model took into account the immortal time bias, by considering VAP as time-dependent covariable [32]. The discrepancy between the lower mortality rate in SARS-CoV-2 patients with VAP, as compared with those with no VA-LRTI (Additional file 1: Table 2), and the adjusted hazard ratio showing an increased risk for mortality in the former than in the latter group (Fig. 2 A) is explained by the immortal time bias in the calculation of actual mortality rate.

Some limitations of this study should be acknowledged. First, coVAPid cohort was retrospective. However, VAP was prospectively identified in all centers. Further, the presence of new infiltrate on chest X-ray was evaluated by at least two physicians. Second, the study was performed in Europe, and the results may not be generalized to other world regions. Third, number of patients was relatively small in some subgroups with VAP or VAT, and our study might have been underpowered to detect differences regarding some secondary outcomes. Fourth, in spite of careful adjustment, our analysis might have missed some residual confounders. Fifth, the interpretation of chest-x ray is a difficult task in patients with pneumonia or ARDS. In spite of evaluation of chest-X ray by at least two physicians to confirm the presence of new infiltrate, some patients with VAP could have been misclassified in the VAT group. Further, some patients with VAT (12.6%) developed subsequent VAP. However, we repeated the analysis on the relationship between VAP and mortality, including VAT patients and the results did not differ (Additional file 1: Fig. 2). Sixth, no information was collected on timing of appropriate initial antibiotic treatment. Finally, patients were only followed-up until day 28. Therefore, outcomes at day 60, or day 90 could not be evaluated.

## Conclusions

To conclude, our results suggest that VAP is associated with increased 28-day mortality rate and longer duration of mechanical ventilation and ICU length of stay in SARS-CoV-2 patients. However, SARS-CoV-2 infection, compared with influenza or no viral infection, has no significant impact on the relationship between VAP and 28-day mortality, or  on the relationship between VAP and duration of mechanical ventilation, and  ICU length of stay. Further studies are needed to confirm our findings.

## Supplementary Information

**Additional file 1**. Online supplementay data.

## Data Availability

All data needed to evaluate the conclusions in this Article are present and tabulated in the main text or the appendix. This article is the result of an original retrospective cohort. For individual de-identified raw data that underlie the results reported in this article, please contact the corresponding author.

## Abbreviations

COVID: Coronavirus disease
HR: Hazard ratio
ICU: Intensive care unit
MV: Mechanical ventilation
SARS-CoV-2: Severe acute respiratory syndrome Coronavirus 2
VA-LRTI: Ventilator-associated respiratory tract infection
VAP: Ventilator-associated pneumonia
VAT: Ventilator-associated tracheobronchitis
