# Supplementary material for: Relationship between ventilator-associated pneumonia and mortality in COVID-19 patients: a planned ancillary analysis of the coVAPid cohort
Source: Crit Care. 2021 May 25;25:177. doi: 10.1186/s13054-021-03588-4 (PMC8146175; doi:10.1186/s13054-021-03588-4)
Supplement: Supplementary file 1 — Additional file 1. Online supplementay data. [file 13054_2021_3588_MOESM1_ESM.docx]

**Online Supplementary Material**

**e-Table 1. Patient characteristics at ICU admission according to disease group, and ventilator-associated lower respiratory tract infections**

|  | SARS-CoV-2 | | | | Influenza | | | | No viral infection | | | |
| --- | --- | --- | --- | --- | --- | --- | --- | --- | --- | --- | --- | --- |
|  | | **No VALRTI**  **n=281** | **VAT**  **n=82** | **VAP**  **n=205** | | **No VALRTI**  **n=336** | **VAT**  **n=39** | **VAP**  **n=107** | | **No VALRTI**  **N=393** | **VAT**  **n=46** | **VAP**  **n=87** |
| Age, years^a^ | | 64 (56 to 73) | 64 (55 to 71) | 62 (53 to 70) | | 62 (54 to 71) | 61 (48 to 72) | 61 (50 to 70) | | 66 (56 to 74) | 64 (56 to 75) | 65 (52 to 72) |
| Men | | 185 (65.8) | 58 (70.7) | 164 (80.0) | | 197 (58.8) | 30 (76.9) | 71 (66.4) | | 258 (65.8) | 32 (71.1) | 63 (72.4) |
| Body mass index, kg/m^2 b^ | | 29 (26 to 34) | 28 (25 to 32) | 29 (26 to 34) | | 27 (23 to 32) | 28 (24 to 31) | 28 (23 to 32) | | 26 (23 to 31) | 26 (24 to 32) | 27 (23 to 32) |
| Severity scores | |  |  |  | |  |  |  | |  |  |  |
| SAPS II^c^ | | 41 (32 to 58) | 42 (34 to 54) | 40 (32 to 52) | | 51 (39 to 65) | 47 (38 to 58) | 50 (39 to 60) | | 56 (42 to 68) | 51 (40 to 61) | 56 (41 to 68) |
| SOFA score^d^ | | 6 (3 to 8) | 6 (4 to 9) | 6 (3 to 9) | | 8 (6 to 11) | 9 (5 to 11) | 9 (6 to 11) | | 8 (5 to 11) | 9 (6 to 11) | 9 (6 to 12) |
| Comorbidity scores | | 3 (1 to 4) | 3 (2 to 4) | 3 (1 to 3) | | 4 (2 to 5) | 3 (2 to 5) | 3 (1 to 4) | | 4 (2 to 6) | 4 (2 to 6) | 3 (2 to 5) |
| MacCabe classification | |  |  |  | |  |  |  | |  |  |  |
| Non-fatal | | 220 (84.9) | 75 (92.6) | 180 (88.7) | | 226 (71.7) | 26 (66.7) | 72 (70.6) | | 225 (62.0) | 33 (75.0) | 57 (69.5) |
| Fatal < 5 years | | 35 (13.5) | 6 (7.4) | 21 (10.3) | | 74 (23.5) | 13 (33.3) | 27 (26.5) | | 106 (29.2) | 8 (18.2) | 23 (28.0) |
| Fatal < 1 year | | 4 (1.5) | 0 (0.0) | 2 (1.0) | | 15 (4.8) | 0 (0.0) | 3 (2.9) | | 32 (8.8) | 3 (6.8) | 2 (2.4) |
| Charlson Comorbidity Index^e^ | |  |  |  | |  |  |  | |  |  |  |
| Chronic diseases | |  |  |  | |  |  |  | |  |  |  |
| Diabetes mellitus | | 76 (27.0) | 25 (30.5) | 67 (33.2) | | 78 (23.6) | 10 (26.3) | 16 (15.1) | | 105 (27.1) | 12 (26.1) | 15 (17.4) |
| Chronic renal failure | | 21 (7.6) | 3 (3.7) | 9 (4.5) | | 26 (7.8) | 2 (5.3) | 11 (10.5) | | 41 (10.5) | 2 (4.3) | 2 (2.3) |
| Cardiovascular disease | | 55 (19.7) | 24 (29.6) | 24 (12.0) | | 77 (23.3) | 11 (28.9) | 29 (27.1) | | 99 (25.6) | 11 (23.9) | 24 (27.9) |
| Chronic heart failure | | 10 (3.6) | 4 (5.0) | 7 (3.5) | | 23 (6.9) | 4 (10.5) | 10 (9.4) | | 43 (11.1) | 4 (8.7) | 3 (3.5) |
| COPD | | 20 (7.2) | 5 (6.2) | 12 (6.0) | | 90 (27.2) | 12 (31.6) | 27 (25.5) | | 73 (18.8) | 8 (17.4) | 17 (19.8) |
| Chronic respiratory failure | | 14 (5.0) | 1 (1.2) | 5 (2.5) | | 48 (14.5) | 8 (21.1) | 11 (10.4) | | 42 (10.9) | 1 (2.2) | 6 (7.0) |
| Cirrhosis | | 5 (1.8) | 2 (2.5) | 1 (0.5) | | 15 (4.5) | 0 (0.0) | 1 (0.9) | | 30 (7.8) | 2 (4.3) | 4 (4.7) |
| Immunosuppression | | 31 (11.1) | 3 (3.7) | 18 (9.0) | | 79 (23.7) | 7 (17.9) | 21 (19.6) | | 93 (24.0) | 8 (17.8) | 16 (18.4) |
| Active smoking | | 14 (5.0) | 4 (4.9) | 11 (5.5) | | 102 (30.8) | 11 (28.9) | 36 (33.6) | | 105 (27.1) | 11 (23.9) | 21 (24.4) |
| Alcohol abuse | | 15 (5.4) | 6 (7.5) | 13 (6.5) | | 65 (19.7) | 4 (10.5) | 16 (15.0) | | 96 (24.8) | 10 (21.7) | 26 (30.2) |
| Location before ICU admission | |  |  |  | |  |  |  | |  |  |  |
| Home | | 132 (47.0) | 43 (52.4) | 96 (46.8) | | 204 (60.9) | 21 (53.8) | 50 (46.7) | | 207 (52.7) | 20 (43.5) | 38 (43.7) |
| Hospital ward | | 120 (42.7) | 27 (32.9) | 68 (33.2) | | 105 (31.3) | 13 (33.3) | 39 (36.4) | | 170 (43.3) | 21 (45.7) | 39 (44.8) |
| Another ICU | | 29 (10.3) | 12 (14.6) | 41 (20.0) | | 26 (7.8) | 5 (12.8) | 18 (16.8) | | 16 (4.1) | 5 (10.9) | 10 (11.5) |
| Admission category | |  |  |  | |  |  |  | |  |  |  |
| Medical | | 281 (100.0) | 82 (100.0) | 204 (99.5) | | 335 (99.7) | 38 (97.4) | 107 (100.0) | | 350 (89.1) | 36 (78.3) | 81 (93.1) |
| Surgical | | 0 (0.0) | 0 (0.0) | 0 (0.0) | | 0 (0.0) | 0 (0.0) | 0 (0.0) | | 13 (3.3) | 3 (6.5) | 1 (1.1) |
| Trauma | | 0 (0.0) | 0 (0.0) | 1 (0.5) | | 1 (0.3) | 1 (2.6) | 0 (0.0) | | 30 (7.6) | 7 (15.2) | 5 (5.7) |
| Recent hospitalization (<3 months) | | 31 (11.1) | 3 (3.7) | 10 (4.9) | | 58 (17.4) | 5 (13.2) | 9 (8.4) | | 119 (30.4) | 8 (17.4) | 21 (24.1) |
| Recent antibiotic treatment (<3 months) | | 48 (17.1) | 9 (11.0) | 17 (8.3) | | 76 (22.9) | 4 (10.5) | 15 (14.0) | | 81 (20.7) | 6 (13.0) | 16 (18.4) |
| Causes for ICU admission | |  |  |  | |  |  |  | |  |  |  |
| Shock | | 219 (78.8) | 51 (62.2) | 128 (64.0) | | 247 (75.1) | 28 (71.8) | 74 (69.2) | | 184 (48.0) | 22 (47.8) | 38 (44.2) |
| Acute respiratory failure | | 264 (94.0) | 67 (82.7) | 190 (92.7) | | 299 (89.3) | 37 (94.9) | 97 (91.5) | | 211 (55.5) | 19 (41.3) | 49 (56.3) |
| ARDS | | 181 (65.1) | 55 (68.8) | 150 (73.2) | | 140 (42.9) | 20 (51.3) | 60 (57.7) | | 50 (13.3) | 7 (15.2) | 15 (17.2) |
| Neurological failure | | 14 (5.1) | 3 (3.9) | 9 (4.5) | | 45 (13.9) | 8 (21.1) | 16 (15.5) | | 143 (37.9) | 16 (34.8) | 32 (37.2) |
| Cardiac arrest | | 3 (1.1) | 0 (0.0) | 0 (0.0) | | 21 (6.5) | 1 (2.6) | 3 (2.9) | | 66 (17.6) | 6 (13.0) | 12 (14.0) |
| Acute kidney injury | | 58 (21.2) | 5 (6.6) | 33 (16.7) | | 84 (26.2) | 14 (37.8) | 35 (34.0) | | 105 (28.2) | 9 (19.6) | 22 (25.3) |

Values are as number (%) or median (interquartile range).

McCabe classification of comorbidities and likelihood of survival, likely to survive > 5 years, 1-5 years, <1 year; Chronic renal failure, KDOQI CKD classification stage 4 or 5 (creatinine clearance < 30 ml/mn); Chronic heart failure, NYHA class III or IV; Heart disease, ischemic heart disease or atrial fibrillation; Cirrhosis, Child-Pugh score B or C; Immunosuppression if haematological malignancy, allogenic stem cell transplant, organ transplant, HIV or immunosuppressive drugs; More than one cause for ICU admission is possible.

| e-Table 2. Description of outcomes of ventilator-associated lower respiratory tract infections | | | | | | | | | | | | |
| --- | --- | --- | --- | --- | --- | --- | --- | --- | --- | --- | --- | --- |
|  | **SARS-CoV-2** | | | | **Influenza** | | | | **No viral infection** | | | |
|  | | **No VALRTI**  **n=281** | **VAT**  **n=82** | **VAP**  **n=205** | | **No VALRTI**  **n=336** | **VAT**  **n=39** | **VAP**  **n=107** | | **No VALRTI**  **N=393** | **VAT**  **n=46** | **VAP**  **n=87** |
| MV duration, days | | 10(7 to 15) | 18 (14 to 25) | 21 (14 to 28) | | 8 (5 to 13) | 17 (8 to 28) | 22 (14 to 28) | | 6 (4 to 11) | 20 (13 to 26) | 19 (12 to 26) |
| Ventilator-free days | | 11 (0 to 20) | 7 (0 to 13) | 1 (0 to 10) | | 17 (0 to 22) | 6 (0 to 17) | 2 (0 to 11) | | 17 (0 to 23) | 4 (0 to 13) | 3.0 (0 to 15) |
| ICU length of stay, days | | 13 (9 to 19) | 21 (15 to 28) | 24 (17 to 28. | | 11 (7 to 18) | 24 (11 to 28) | 26 (17 to 28) | | 9 (6 to 15) | 26 (16 to 28) | 22.0 (16 to 28) |
| ICU-free days | | 4 (0 to 15) | 0 (0 to 8) | 0 (0 to 6) | | 11 (0 to 19) | 0 (0 to 12) | 0 (0 to 4) | | 10 (0 to 20) | 0 (0 to 10) | 0 (0 to 9) |
| ICU mortality | | 95 (33.8) | 17 (20.7) | 52 (25.4) | | 87 (25.9) | 9 (23.1) | 29 (27.1) | | 141 (35.9) | 11 (23.9) | 21 (24.1) |
| D28 mortality | | 96 (34.2) | 17 (20.7) | 53 (25.9) | | 92 (27.4) | 9 (23.1) | 31 (29.0) | | 146 (37.2) | 13 (28.3) | 23 (26.4) |

Data are presented as n (%) or median (interquartile range).

Abbreviations: MV, Mechanical Ventilation; ICU, Intensive Care Unit; VA-LRTI, ventilator-associated respiratory tract infection; VAT, ventilator-associated tracheobronchitis; VAP, ventilator-associated pneumonia.

**e-Figure 1. Cumulative incidence of 28-day mortality (A), extubation alive (B) and discharge alive (C) according to study groups**

**A**


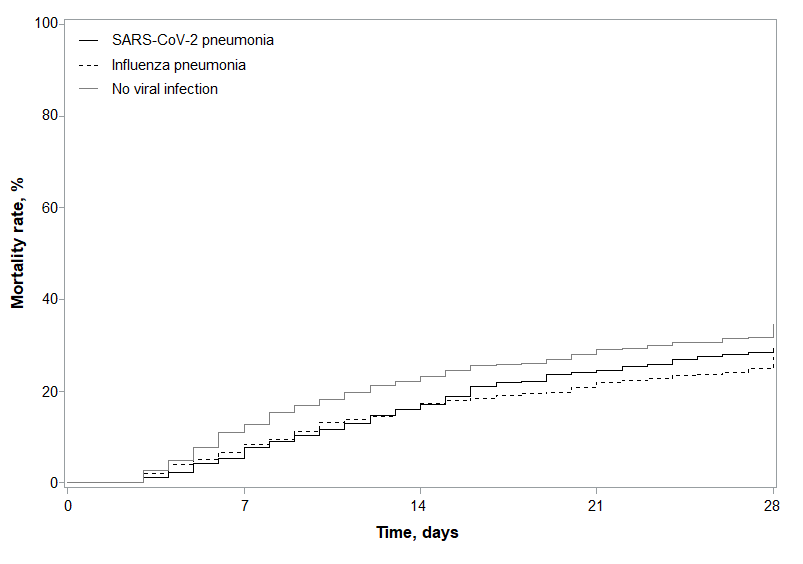


**B**


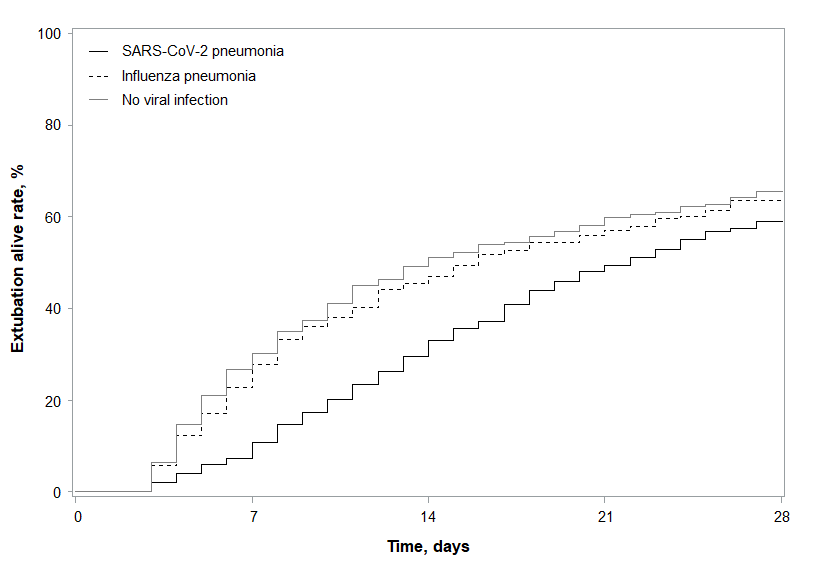


**C**


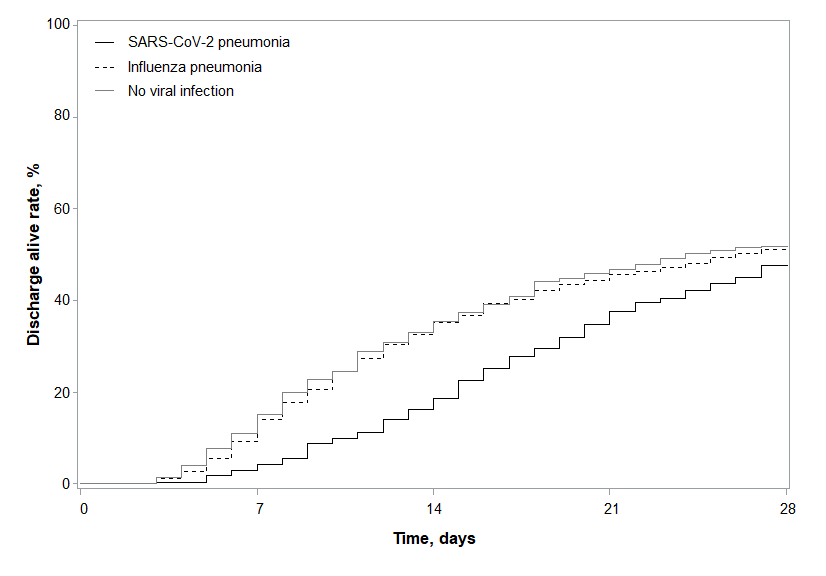


**e-Figure 2 Association between ventilator-associated lower respiratory tract infections (VAP and VAT) with outcomes according to study subgroups**


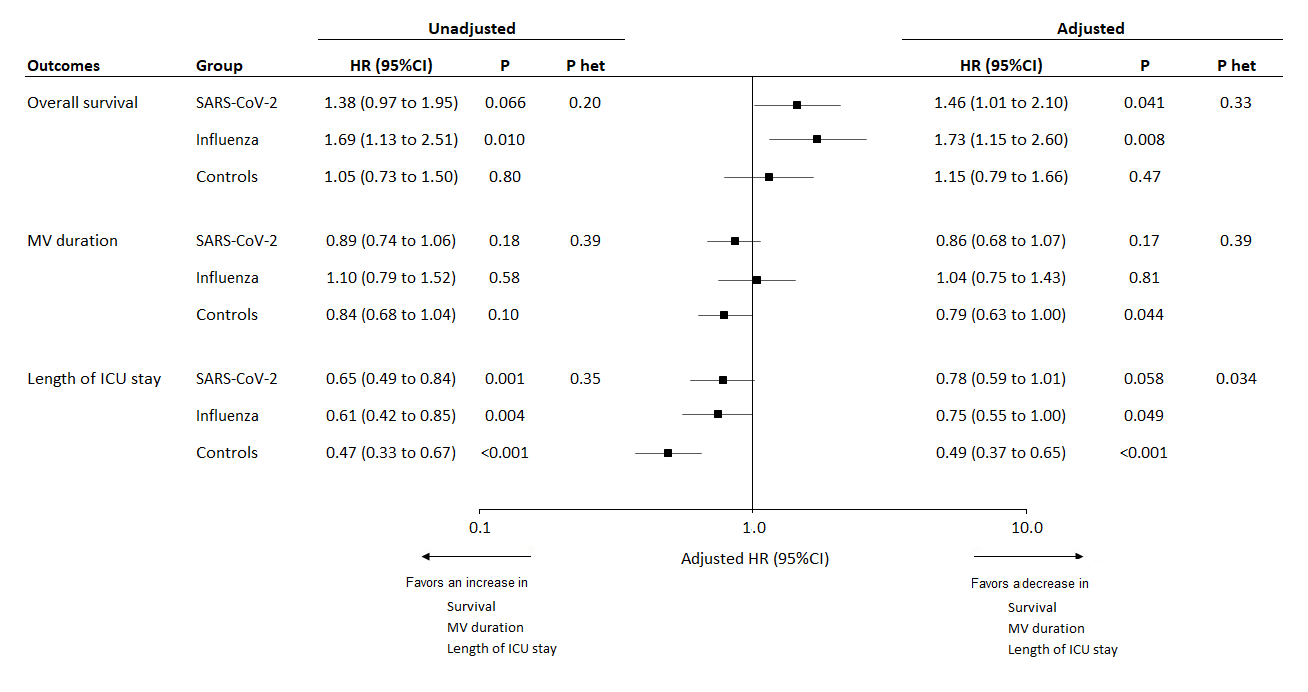


HRs were calculated using Cox’s proportional hazard models, with a cause-specific hazard approach for MV duration and length of ICU stay, with extubation alive and ICU discharge as events of interest and death as competing event. In all Cox’s models, VA-LRTI was considered as a time dependent binary covariable (No VA-LRTI vs. VA-LRTI (VAT or VAP)). Adjusted HRs were calculated by including age, gender, simplified acute physiology score II, Charlson score, MacCabe classification, shock, and acute respiratory distress syndrome as pre-specified covariables into each Cox’s model.

A HR>1 indicates a decrease in survival duration (i.e. an increased risk for mortality), MV duration (i.e. an increased risk for extubation alive) and ICU length of stay (i.e. an increased risk for discharge alive) and a HR<1 indicates an increase in survival duration (i.e. a decreased risk for mortality), MV duration (i.e. a decreased risk for extubation alive) and ICU length of stay (i.e. a decreased risk for discharge alive). Note that the event of interest for survival is a pejorative event (death), whereas for MV duration and ICU length of stay, the event of interest is a positive event (extubation or discharge alive). Consequently, the detrimental effect of VA-LTRI was associated with a HR>1 for overall survival, but was associated with a HR<1 for MV duration and ICU length of stay.

Abbreviations: HR, hazard ratio; ICU, intensive care unit; MV, mechanical ventilation; VA-LRTI, ventilator-associated respiratory tract infection; VAP, ventilator-associated pneumonia; VAT, ventilator-associated tracheobronchitis.
